# Strategies for recruitment of adolescent girls into physical activity programmes: a systematic review protocol

**DOI:** 10.12688/hrbopenres.13834.1

**Published:** 2024-02-16

**Authors:** Tanya O'Brien, Catherine D Darker, David Mockler, Emer M Barrett

**Affiliations:** 1 Physiotherapy, The University of Dublin Trinity College, Dublin, Leinster, Ireland; 2 Public Health & Primary Care, The University of Dublin Trinity College, Dublin, Leinster, Ireland; 3 School of Medicine, The University of Dublin Trinity College, Dublin, Leinster, Ireland

**Keywords:** physical activity, adolescents, adolescent girls, exercise, recruitment, enrolment

## Abstract

**Background:**

Physical activity is essential for youth physical and mental health, yet just 15% of adolescent girls versus 22% of adolescent boys worldwide meet the World Health Organization guideline of 60 minutes of moderate-to-vigorous physical activity per day. As activity patterns established in adolescence often persist into adulthood, there is a looming risk of adverse health outcomes due to insufficient physical activity. A persistent challenge faced by physical activity providers, however, is recruiting adolescent girls into their programmes. This systematic review will quantitatively synthesise existing knowledge surrounding the recruitment of adolescent girls into physical activity interventions and aims to provide evidence-based recommendations for optimal recruitment practices.

**Methods:**

Five electronic databases will be searched to identify randomised controlled trials of physical activity interventions for adolescent girls worldwide. Hand-searches of reference lists of included randomised controlled trials, relevant systematic reviews, and author publications will also be conducted. Data will be extracted regarding study, participant, and intervention characteristics, pre-determined recruitment goals, recruitment strategies employed, and the number of participants screened, eligible, approached, randomised, and retained. Outcomes will include whether pre-determined recruitment goals were met, recruitment rate, and any adapted or extended recruitment measures required mid-study. Descriptive statistics, including the median recruitment rate for all included studies, will be calculated and stratified by subgroups such as the type or setting of physical activity. Recruitment strategies will be categorised, and the percentage of studies using each category, along with their associated recruitment rates, will be documented. If sufficient studies are found, multi-variable regression will be employed to determine if the use of a specific recruitment strategy increases enrolment.

**Discussion:**

Application of the findings of this review by physical activity programme providers may enhance their recruitment processes, potentially increasing teenage girls’ physical activity enrolment worldwide.

**Systematic review registration:**

PROSPERO CRD#42023475858

## Introduction

Physical activity (PA) is essential for youth physical and mental health, and yet just 15% of adolescent girls versus 22% of adolescent boys worldwide meet the World Health Organization (WHO) guideline of 60 minutes of moderate-to-vigorous physical activity per day ^1^. There is a recognised gender gap when it comes to adolescent PA participation ^2, 3^, with a recent analysis from 146 countries showing that PA participation in girls was 7% lower than boys ^3^. Regular exercise improves self-esteem, academic performance, cardiovascular health, and muscular fitness while reducing anxiety, depression, diabetes, cardiovascular disease, and obesity risk ^4^. Furthermore, PA habits established in adolescence often endure into adulthood, putting this cohort of inactive adolescent girls at risk of adverse health outcomes due to insufficient physical activity ^5^.

In 2013, global physical inactivity cost healthcare systems internationally $ 53.8 billion due to the management of lifestyle diseases such as coronary heart disease, stroke, and type 2 diabetes ^6^. In addition to direct healthcare costs, there are indirect annual costs related to decreased productivity ($13.7 billion), not to mention the health-related impact of 13.4 million disability-adjusted life years related to physical inactivity ^6^. These estimates are likely to be even higher in 2023, as childhood and adult obesity levels are on the rise, and worldwide obesity levels have tripled since 1975 ^7^. In 2016, 340 million children and adolescents aged 5–19, or just over 18%, were overweight or obese ^7^. Fortunately, increasing awareness of the growing crisis of inactivity, and in adolescent girls specifically, has resulted in the development of new, generally well-received PA programmes based on state-of-the-art behaviour change theories and frequently involving adolescent girls in the design or evaluation of these programmes ^8– 11^.

A persistent challenge for PA programme providers, however, is recruiting adolescent girls to join their programmes ^8, 12– 14^, with reduced interest from potential participants being cited as one of the main barriers to offering after-school extracurricular activities ^9^. Girls PA programme providers, for example, report recruiting only 10–29% of eligible adolescent girls even with multiple recruitment rounds ^8, 10^, note that adjustments to their methodology were required to improve recruitment ^11, 15^, or end up enrolling fewer participants than intended when compared with their pre-determined recruitment goals ^16, 17^.

Currently, there is a dearth of quantitative research internationally regarding effective recruitment practices for adolescent girls into PA programmes. Recent qualitative research has examined adolescent girls’ perceptions of PA, the types of PA programmes they are looking for, and facilitators and barriers to their participation ^18– 21^. Individual study authors list the recruitment strategies employed in their programmes, such as presentations from researchers or teachers ^22– 24^, taster sessions in advance of signing up for the entire programme ^25^, informational emails sent by schools ^24^, posters ^24^, flyers ^26^, invitational letters to parents ^23^, incentives for participation ^23^, social media ^26, 27^, and more.

Quantitative evaluations of the effectiveness of these recruitment strategies in this demographic, however, are lacking. There is very little evidence to guide programme providers on which strategies can increase enrolment and whether there are differences in recommended strategies based on subgroups such as older or younger adolescent age, urban versus rural populations, or the setting of the physical activity programme such as during physical education class, after-school on school grounds, or in the community. Therefore, this systematic review will present median recruitment rates for enrolling adolescent girls into PA programmes, synthesise existing knowledge surrounding strategies employed to recruit adolescent girls into PA programmes, and attempt to quantitatively determine which recruitment strategies are most successful with this population.

## Protocol

### Methods/design

This protocol follows the Preferred Reporting Items for Systematic Reviews and Meta-Analysis – Protocol (PRISMA-P) guidelines and is reported utilising the PRISMA-P checklist ^28^ (Additional file 1). This review topic has been registered with the PROSPERO database (registration number CRD#42023475858). Should any amendments to this protocol be required, an update to this protocol publication will be submitted.

### Aim

The aims of this systematic review are (i) to establish recruitment rates for adolescent girls into Randomised Controlled Trials (RCTs) of PA programmes, (ii) to synthesise existing knowledge surrounding strategies employed by PA programme providers to enrol adolescent girls into RCTs of PA programmes, and (iii), to quantitatively determine which recruitment strategies are most effective with this cohort, resulting in evidence-based recommendations to inform the recruitment practices of PA providers enrolling adolescent girls into PA intervention RCTs.

### Eligibility criteria

The eligibility criteria for inclusion in this review will be based on the PICOTS Framework (Population, Intervention, Comparison, Outcome, Time frame, Study Type) ^29^.

### Population

Studies that include healthy adolescents who identify as female, aged 10–19, as per the WHO’s definition of adolescents ^30^ will be included. Any recorded socioeconomic status indicators, such as programme locations in areas of deprivation, will be noted for subgroup analyses. All locations worldwide will be included.

Studies that include adolescents who do not identify as female, who are outside the included age range, or who have been identified as a special population due to a health condition will be excluded. Studies that include only overweight or obese cohorts, or only athletic cohorts from specific sports teams will be excluded.

### Interventions

Any PA programmes, defined for the purposes of this review as organised, repeated PA activities, which may be online, community, or school-based programmes that provide adolescent girl-only physical activity opportunities, will be included. Examples of eligible PA programmes might include activities such as running, dance, Zumba, callisthenics, football, weight training, and more. All settings will be included; for example, programmes carried out during school Physical Education classes, programmes offered as extracurricular after-school activities on school grounds, online programmes, or community-based after-school or weekend activities at local facilities. Multi-component programmes, such as those also including nutrition advice, mental health education, or leadership skills, will be included, provided that the primary intervention is physical activity. These will be noted as a potential subgroup for comparison with PA-only opportunities.

The number of adolescent girls Screened (S), Eligible (E), Approached (A), and Randomized (R), as per the SEAR framework ^31^, will be recorded, alongside the number of girls who completed the programme, retention rates, and any pre-determined recruitment goals, which may be sourced from published RCT protocols. Characteristics of the recruitment strategies employed, such as the recruitment modality (e.g. flyers, posters, emails, presentations, or social media) and who carried out the recruitment strategies (e.g. teachers, researchers, community leaders, or coaches) will be noted. A description of any changes to the recruitment process throughout the study will be recorded.

To be included, studies must report either (i) the number of eligible girls they approached, and the number subsequently Screened, Eligible, or Randomised or (ii) a pre-determined recruitment goal and the final number of Screened, Eligible, or Randomised participants.

### Comparison

As only RCTs will be included, all studies will include an individual or cluster-randomised control group. This control group must receive either no physical activity programme or a usual practice physical activity programme such as their typical Physical Education class rather than an enhanced or modified class. Physical activity advice without the provision of an actual physical activity programme will also be accepted as a control group.

### Outcomes

***Primary outcome(s)***

- Characteristics of the recruitment strategies employed
- Binary description of whether the study met or did not meet pre-determined recruitment goals
- Percentage of eligible participants recruited versus approached (recruitment rate)

***Secondary outcome(s)***

- Challenges identified by the research teams regarding recruitment
- Any extra measures or changes to the recruitment process employed mid-study
- Characteristics of the physical activity programme such as setting, physical activity modality, and whether it is a PA-only or multi-faceted programme
- Number and percentage of girls retained until the end of the programme (retention rate)

### Timeframe

There will be no retrospective start date for the date of publication, and included studies may be published until the search date of 3 February 2023. This search will be updated prior to submission of the completed Systematic Review manuscript for publication to ensure any new, relevant articles are included.

### Study type

This review will include primary research studies that implement physical activity programmes or interventions for adolescent girls aged 10–19. Individual and cluster randomised controlled trials (RCTs) will be included, provided that the required quantitative recruitment-related data is provided. The study type was limited to RCTs as RCTs have been designated the ‘gold standard’ in effectiveness research, with higher standards regarding study conduct and reporting ^32^.

Unpublished, pre-print, non-peer-reviewed studies, conference abstracts, systematic reviews, narrative reviews, oral presentations not available in full text, and grey literature will be excluded.

### Information sources and search strategy

The following electronic databases will be included in our search: Embase, Medline, CINAHL, Web of Science, and the Cochrane Library – Central Trial Registry. The search strategy will include database-specific terms, truncations, and synonyms combining ‘adolescent females’, ‘recruitment’, and ‘physical activity’ (Additional File 2).

Where multiple papers exist for a single RCT, the most relevant paper for each unique RCT will be designated the included ‘parent study’. If required, a hand-search of related papers such as study protocols, process evaluations, and trial registers will be retrieved from the publication lists of the first and last authors of the parent study to identify pre-determined recruitment goals, recruitment rate, and recruitment strategies employed. In instances where the recruitment-related data is partially available in existing publications, the research team will contact the study authors by email on two occasions to attempt to retrieve the remaining recruitment data. All relevant related papers will be collated with the parent RCT in Covidence and imported into EndNote for appropriate tracking and referencing.

## Study records

### Data management

Studies will first be imported into EndNote 20.2 Desktop software for reference management, then into Covidence’s online systematic review management software. Duplicates will be removed automatically. Screening, selection, and data extraction will be performed in Covidence. Following this process, quantitative data will be exported for analysis into software such as Microsoft Excel, SPSS, and RevMan 5.4.

### Screening and selection process

Title, abstract, and full-text screening of studies for inclusion/exclusion will be performed independently by two authors in Covidence; discussion will follow to reach consensus should there be any discrepancies between authors. If the required recruitment data for a parent RCT cannot be found in published sources and the study authors do not respond to email or cannot provide the data, the study will be excluded. Any disagreements will be resolved through discussion with the third reviewer.

### Data extraction process

A custom data extraction template, based on the Cochrane Review data extraction template ^33^ and modified to include additional, relevant recruitment-related details will be designed and piloted by TOB. Recruitment methods will be coded into categories; for example, social media advertising, paper flyers, electronic communication with parents, or in-person advertising by researchers or schoolteachers.

Data extracted will include recruitment details such as recruitment strategies, any pre-determined recruitment goals, the number of participants from each category of the SEAR framework ^31^, the recruitment rate, the retention rate, the method of parental consent (i.e. opt-in versus opt-out), and whether the trial met or did not meet their pre-determined recruitment goals. Whether the study authors noted any difficulties with recruitment, whether they employed any adapted or extended recruitment measures mid-study, and a description of these extra recruitment measures will be recorded. Study details such as author(s), year of publication, date(s) and location(s) where the intervention was delivered, the aim of the study, primary outcomes, and study design will be recorded. Population details such as age range, socioeconomic status indicators, and community type (urban versus rural) will also be noted. Recorded details for each intervention will include the setting (e.g. after-school or community-based), the components of the programme (e.g. PA-only or multimodal with leadership skills or other components), the physical activity modality delivered (e.g. dance, running, weight-training), the duration of the programme, the number of sessions per week, the duration of each session, the number of instructors and participants at the first and last sessions, retention rates, and methods of data collection for the studies’ primary outcome(s) (e.g. self-report, accelerometer, objective measurement of weight and body fat percentage).

As this review is not a typical systematic review wherein the effect of the intervention on the outcome could be biased by interpretation (see Quality Assessment section for further details), data will be extracted by one author (TOB) for each included study, followed by a second author performing a duplicate extraction of a randomly selected 10% of studies. If the agreement level between authors is satisfactory, data extraction will continue with one author. Any disagreements will be resolved through discussion with an independent third reviewer. A PRISMA flow diagram will be completed to illustrate the screening process ^34^.

### Quality assessment

Traditional quality assessment is designed to assess each study’s ability to limit the risk of bias regarding the primary outcome(s) of the RCT. In this instance, outcomes such as the impact of the studies’ interventions on physical activity levels or bone mineral density, for example, are the main outcomes, rather than the recruitment data being extracted for this systematic review. Therefore, quality assessment and related assessment of meta-biases was deemed unnecessary for this review given that included studies would not have been randomised or blinded for recruitment-related outcomes. This methodological decision is aligned with similarly styled systematic reviews examining recruitment rates and strategies in health-related RCTs ^35– 38^. Selective reporting within studies of recruitment strategies and related data will be reported.

### Data synthesis

Descriptive statistics, such as median recruitment rate, will be computed for the combined data of all studies to determine the average recruitment rate for all physical activity RCTs within this cohort. Subgroup analysis will then be performed to determine the mean and median recruitment rates categorised by physical activity or trial setting (e.g. school-based, community-based, online). Additional subgroup analyses will explore recruitment rates based on socioeconomic status indicators, urban versus rural locations, physical activity programme type, and older versus younger adolescent age.

Similarly to the categorisation practices used by Reynolds, O’Connor ^35^, recruitment strategies will be categorised by the authors according to common themes (e.g., active vs. passive, school-based, parent-targeted). We will calculate the percentage of studies using each category of recruitment strategies, along with the mean and median recruitment rates when each category of strategies is present. We will document whether studies achieved their predefined recruitment goals, noting strategies employed by successful and unsuccessful cases. Additionally, the use of supplementary recruitment measures, like extended timelines, will be recorded and compared to strategies utilised when such measures were unnecessary using statistical methods.

As per current best-practice recommendations ^33^, if ≥ 10 studies are included, multi-variable regression will be performed to investigate the effect of categorical explanatory variables such as recruitment strategy category, PA programme type, and programme setting on recruitment rate. This would allow us to present evidence-based recommendations based on statistical significance and effect size.

### Analysis of subgroups or subsets

Subgroup analysis will be performed based on the setting of the PA programme, PA programme type, younger adolescent age (10–14), older adolescent age (15–19), and socioeconomic status indicators.

## Discussion

Recruitment of adolescent girls remains a persistent challenge for PA programme providers ^8, 12– 14^, and yet there is a dearth of research examining the recruitment of this demographic into PA programmes or interventions. This quantitative systematic review will complement the recent qualitative reviews examining barriers, facilitators, and perceptions of PA amongst adolescent girls ^18, 20, 39, 40^ and directly address the challenge of recruiting adolescent girls into PA programmes and interventions.

One challenge we expect to encounter is that RCTs may not fully report all details of their recruitment strategies, challenges associated with recruitment, or any extended or extra steps taken to achieve recruitment goals. We hope to mitigate this challenge by contacting study authors for further details as needed and employing inclusion criteria that accept the reporting of either recruitment rate or pre-determined recruitment goals. Furthermore, there are difficulties surrounding the definition of recruitment ‘success’. To these authors’ knowledge, there are no established metrics in this cohort defining what is considered a successful, or even expected, recruitment rate into physical activity programmes outside of meeting or not meeting pre-determined recruitment goals. Metrics of recruitment ‘success’ in other cohorts vary greatly throughout the literature. In cancer survivors, the median recruitment rate into exercise trials has been shown to be 38% ^35^, however this is a specific patient population and may not be representative of adolescent girls. In patients with advanced cancer, the mean recruitment rate into exercise trials was 49% ^41^, but again may not be representative of teenage girls. A systematic review of recruitment into workplace physical activity interventions categorised successful recruitment as a recruitment rate ≥70% ^42^, however, this metric of success was defined for a specific group of mixed gender adults rather than adolescent girls.

Meeting or not meeting pre-determined recruitment goals may be a problematic definition of recruitment success, as these authors surmise that RCTs may change their recruitment strategies, extend their recruitment timeframe, or increase personnel and financial resources mid-study until these goals are met so that power calculations are satisfied. Therefore, this metric might judge most PA RCTs' successful', whereas a different picture may emerge when the recruitment rate is also considered.

Post-analysis, we aim to provide descriptive statistics on projected recruitment rates for adolescent girls' PA RCTs and, where possible, offer evidence-based recommendations for optimal recruitment strategies. These findings will assist programme providers and researchers in evaluating recruitment efficacy and determining the required outreach and recommended strategies to achieve desired sample sizes. Moreover, the dissemination and implementation of evidence-based recruitment recommendations for adolescent girls in PA interventions could substantially increase teenage girls’ PA enrolment worldwide.

## List of abbreviations

CI: Confidence Interval

PA: Physical Activity

PICOTS: Population, Intervention, Comparison, Outcome, Timeframe, Study Type

PRISMA: Preferred Reporting Items for Systematic Reviews and Meta-Analyses

PRISMA-P: Preferred Reporting Items for Systematic Reviews and Meta-Analyses Protocols

RCT: Randomized Controlled Trial

RoB2: Risk of Bias 2

SEAR: Screened, Eligible, Approached, Randomized

WHO: World Health Organization

## Data Availability

No data are associated with this article. ***Reporting guidelines*** Open Science Framework: The PRISMA-P Checklist is registered with the Open Science Framework as “Strategies for recruitment of adolescent girls into physical activity programmes: a systematic review protocol PRISMA-P Checklist”, https://doi.org/10.17605/OSF.IO/YAEW8 ^43^. Data are available under the terms of the Creative Commons Attribution 4.0 International license (CC-BY 4.0).
