# Supplementary material for: Strategies for recruitment of adolescent girls into physical activity programmes: a systematic review protocol
Source: HRB Open Res. 2024 Dec 24;7:6. Originally published 2024 Feb 16. [Version 2] doi: 10.12688/hrbopenres.13834.2 (PMC11528215; doi:10.12688/hrbopenres.13834.2)
Supplement: Peer review history

# Reviewer report: Reviewer response for version 2

**DOI:** 10.21956/hrbopenres.15430.r44752

**Published:** 2025-01-31

**Authors:** Yumi Kim

**Affiliations:** 1 University of Alabama at Birmingham, Birmingham, AL, USA

Thank you for the opportunity to review your submission to HRB Open Research. Engagement in physical activity, along with other health-enhancing behaviors, is a critical topic in medicine. Adolescent females represent a key subgroup who need to develop health these behaviors for lifelong health management. Effectively recruiting and engaging them in physical activity interventions is essential for fostering sustainable participation. While the protocol appears to be well-developed with detailed consideration and methodological rigor, I offer minor suggestions to improve clarity and information presentation. I look forward to reading the findings of this review.

**Aims**

 While the focus on recruitment rates is well articulated, I suggest broadening the scope of the review to include additional response rates, such as adherence to the intervention and retention to follow-up. This expansion could be reflected in the aims section. Currently, Aim 1 appears to be solely focused on recruitment rates, but in the "Intervention" subsection, the authors indicate an intent to report adherence and retention rates. To clarify this point, Aim 1 could be reframed to encompass overall response rates, including recruitment, adherence, and retention. If you are willing to take this suggestion above, to enhance distinction, Aim 2 could specifically address the synthesis of the recruitment strategies and identification of successful recruitment strategies, while Aim 3 could concentrate on strategies that enhance adherence and retention. This revision would enrich the review’s narrative and provide a more comprehensive understanding of program effectiveness beyond recruitment alone.

**Protocol**

 I recommend adding a PRISMA-P flowchart (or diagram) in addition to the checklist, as a visual supplement can offer a clear snapshot of the study selection process.

**Population**

 The last two sentences of this section could be revised for clarity. For instance, you may provide the listed conditions as examples, such as: "... identified as a special population due to a health condition will be excluded, such as overweight, obesity, or other relevant health conditions." Then, I would separate the exclusion of athletic cohorts into a new sentence and explicitly state the justification for this exclusion. Additionally, if clinical trials including mixed-gender programs (boys and girls together) are to be excluded from the final synthesis, this should be explicitly stated.

**PICOTS - Timeframe**

 I appreciate that the study is structured using the PICOTS framework. To enhance clarity, I suggest specifying the duration of interventions under this subheading. It would be helpful to define the minimum criteria for an intervention. For example, if a program is delivered only once or over a brief period (e.g., one week), followed by post-intervention measures, would it still be included in the review?

Is the study design appropriate for the research question?

Yes

Is the rationale for, and objectives of, the study clearly described?

Yes

Are sufficient details of the methods provided to allow replication by others?

Yes

Are the datasets clearly presented in a useable and accessible format?

Not applicable

Reviewer Expertise:

Physical activity intervention design and implementation; Health promotion for people with disabilities; Systematic review methods.

I confirm that I have read this submission and believe that I have an appropriate level of expertise to confirm that it is of an acceptable scientific standard.

# Reviewer report: Reviewer response for version 1

**DOI:** 10.21956/hrbopenres.15158.r42534

**Published:** 2024-10-31

**Authors:** Emma Pollock

**Affiliations:** 1 The University of Newcastle School of Medicine and Public Health, Callaghan, New South Wales, Australia

**Title:** Strategies for recruitment of adolescent girls into physical activity programmes: a systematic review protocol

**Summary: **Thank you for the opportunity to review this well-written manuscript. This systematic review protocol will examine recruitment strategies for engaging adolescent girls in physical activity interventions, addressing a critical need as only 15% meet the WHO's physical activity guideline. By synthesising data from randomised controlled trials, it will assess recruitment rates, strategies, and participant retention. Descriptive statistics will explore the effectiveness of various recruitment methods. The findings aim to guide program providers on optimising recruitment, ultimately supporting more girls in meeting physical activity recommendations that can lead to improved lifelong health.

 To improve the quality of the manuscript, the authors could consider the following:

- Although this information may not be available in many studies, including the distribution setting of recruitment strategies (e.g., There may be differences in effectiveness depending on whether a flyer is handed directly to a student by a teacher during class time versus distributed at a P&C meeting) and the intended target audience of the recruitment material (e.g., adolescent girls directly or their parents/caregivers) as key characteristics could provide valuable insights. This approach would help identify which environments (e.g., schools, community, online platforms) and target audiences are most effective in engaging adolescent girls in physical activity programs.
- While it may be beyond the scope of this study, it could be valuable to identify whether any specific retention strategies were implemented. Although the secondary aim includes examining any additional measures or adjustments to recruitment, understanding whether retention strategies were planned—particularly for longer-duration projects—could provide further insights into sustaining participant engagement.

Is the rationale for, and objectives of, the study clearly described? Yes

 Is the study design appropriate for the research question? Yes

 Are sufficient details of the methods provided to allow replication by others? Yes

 Are the datasets clearly presented in a useable and accessible format? Yes

Is the study design appropriate for the research question?

Yes

Is the rationale for, and objectives of, the study clearly described?

Yes

Are sufficient details of the methods provided to allow replication by others?

Yes

Are the datasets clearly presented in a useable and accessible format?

Yes

Reviewer Expertise:

Physical activity in children and adolescents; girls' physical activity levels and sport participation; School- and community-based physical activity interventions; Physical Education (PE); implementation and sustainment of physical activity programs.

I confirm that I have read this submission and believe that I have an appropriate level of expertise to confirm that it is of an acceptable scientific standard.

# Response

**Published:** 2024-12-16

**Authors:** Tanya O'Brien

**Affiliations:** Physiotherapy, The University of Dublin Trinity College, Dublin, Leinster, Ireland

Dear Reviewer, thank you for your very helpful and insightful comments. We have updated our manuscript accordingly, with the following revisions.

**Reviewer 2:**

**Comment 1: **

- To improve the quality of the manuscript, the authors could consider the following: Although this information may not be available in many studies, including the distribution setting of recruitment strategies (e.g., There may be differences in effectiveness depending on whether a flyer is handed directly to a student by a teacher during class time versus distributed at a P&C meeting) and the intended target audience of the recruitment material (e.g., adolescent girls directly or their parents/caregivers) as key characteristics could provide valuable insights. This approach would help identify which environments (e.g., schools, community, online platforms) and target audiences are most effective in engaging adolescent girls in physical activity programs.

***Response:** Thank you for this very helpful comment. As you have suggested, we will make note of the recruitment strategy setting in hopes of identifying which environments are most effective. The manuscript has been updated accordingly.*

**Comment 2:**

- While it may be beyond the scope of this study, it could be valuable to identify whether any specific retention strategies were implemented. Although the secondary aim includes examining any additional measures or adjustments to recruitment, understanding whether retention strategies were planned—particularly for longer-duration projects—could provide further insights into sustaining participant engagement.

***Response:** Thank you for this very helpful comment. While we will be reporting on retention as a secondary outcome, strategies to maintain retention are outside the scope of this current review. Depending on our findings, however, we will likely be including recommendations for future research around retention, as you have suggested.*

# Reviewer report: Reviewer response for version 1

**DOI:** 10.21956/hrbopenres.15158.r38574

**Published:** 2024-05-11

**Authors:** Joey Murphy

**Affiliations:** 1 Centre for Exercise, Nutrition and Health Sciences, School for Policy Studies, University of Bristol, Bristol, UK

Thank you for inviting me to review your work. This is well written and there is clear value in completing this systematic review. Some minor comments/suggestions below. Best of luck with this work. 

 Abstract and Introduction

- The authors state the WHO guideline as “60 minutes of MVPA per day”. I realise this is how it is quoted in the WHO news release, however, would recommend using an “average of 60 minutes of MVPA per day” so that it aligns with the current WHO guidelines.

Protocol

- Can the authors explain why they are excluding studies that include only overweight or obese cohorts? These would be seen as “hard to reach” and could gain the greatest benefit in physical activity engagement – so it seems like a missed opportunity.
- The secondary outcomes are interested in highlighting challenges identified regarding recruitment, which is great. However, it would also be useful to highlight recruitment facilitators noted in studies as this can help share good practice. 
- Will Boolean operators be used to develop the final search strategy? If so, I would encourage adding to the protocol so it is clear how the search strategy will be developed. This may be available in Additional File 2, but I can’t access.

Is the study design appropriate for the research question?

Yes

Is the rationale for, and objectives of, the study clearly described?

Yes

Are sufficient details of the methods provided to allow replication by others?

Yes

Are the datasets clearly presented in a useable and accessible format?

Not applicable

Reviewer Expertise:

Physical activity and public health, implementation research, systematic literature review methods.

I confirm that I have read this submission and believe that I have an appropriate level of expertise to confirm that it is of an acceptable scientific standard.

# Response

**Published:** 2024-12-16

**Authors:** Tanya O'Brien

**Affiliations:** Physiotherapy, The University of Dublin Trinity College, Dublin, Leinster, Ireland

Dear Reviewer, thank you for your very helpful and insightful comments. We have updated our manuscript accordingly, with the following revisions.

**Reviewer 1:**

**Comment 1: **

- The authors state the WHO guideline as “60 minutes of MVPA per day”. I realise this is how it is quoted in the WHO news release, however, would recommend using an “average of 60 minutes of MVPA per day” so that it aligns with the current WHO guidelines.

*Response: Thank you for this very helpful comment. We have updated the manuscript as you have suggested.*

**Protocol**

**Comment 2:**

- Can the authors explain why they are excluding studies that include only overweight or obese cohorts? These would be seen as “hard to reach” and could gain the greatest benefit in physical activity engagement – so it seems like a missed opportunity.

*Response: Thank you for your insightful comment. We agree that this cohort can be challenging to engage and would benefit from physical activity initiatives. For this review, we have focused on the 'typical' adolescent girl, which may include those with overweight or obesity, but have excluded cohorts that exclusively comprise 'special populations' to limit the project's scope.*

**Comment 3: **

- The secondary outcomes are interested in highlighting challenges identified regarding recruitment, which is great. However, it would also be useful to highlight recruitment facilitators noted in studies as this can help share good practice. 

*Response: This is an excellent suggestion and we have updated the protocol accordingly.*

**Comment 4: **

- Will Boolean operators be used to develop the final search strategy? If so, I would encourage adding to the protocol so it is clear how the search strategy will be developed. This may be available in Additional File 2, but I can’t access.

*Response: Thank you for highlighting this oversight regarding access to the search strategy. Boolean operators were used in its development, and it was previously included in Additional File 2. However, as HRB Open no longer publishes supplementary material, we have updated the Open Science Framework repository with the search strategy with this now included. The link to this repository has been included here for your convenience:* https://doi.org/10.17605/OSF.IO/YAEW8 .**
